# Insularization drives physiological condition of Amazonian dung beetles

**DOI:** 10.1002/ece3.10772

**Published:** 2023-12-06

**Authors:** Leonardo Vilas‐Bôas M. P. de Cerqueira, Daniel González Tokman, César M. A. Correa, Danielle Storck‐Tonon, Mario Cupello, Carlos A. Peres, Renato Portela Salomão

**Affiliations:** ^1^ Programa de Pós‐Graduação em Ecologia Instituto Nacional de Pesquisas da Amazônia Manaus Brazil; ^2^ Red de Ecoetología Instituto de Ecología A. C. Xalapa Mexico; ^3^ Laboratório de Bioecologia de Scarabaeoidea (Scaralab) Universidade Estadual de Mato Grosso do Sul Aquidauana Brazil; ^4^ Laboratório de Zoologia, CPEDA Universidade do Estado de Mato Grosso Tangará da Serra Brazil; ^5^ Departamento de Zoologia, Laboratório de Sistemática e Bioecologia de Coleoptera Universidade Federal do Paraná Curitiba Brazil; ^6^ School of Environmental Sciences University of East Anglia Norwich UK; ^7^ Facultad de Estudios Superiores Iztacala Universidad Nacional Autónoma de México Tlalnepantla de Baz Mexico

**Keywords:** Amazon rainforest, environmental impact, habitat fragmentation, landscape ecology, Scarabaeinae

## Abstract

The fragmentation and degradation of otherwise continuous natural landscapes pose serious threats to the health of animal populations, consequently impairing their fitness and survival. While most fragmentation ecology studies focus on habitat remnants embedded withinn terrestrial matrices, the effects of true insularization remains poorly understood. Land‐bridge islands created by major dams leads to habitat loss and fragmentation, negatively affecting terrestrial biodiversity. To assess the effects of insularization, we conducted a study on the key aspects of dung beetle physiological condition and body size throughout the Balbina Hydroelectric Reservoir located in the Central Amazon. We assessed these traits at the population and assemblage levels, collecting dung beetles from both forest islands and continuous forest areas while analyzing various landscape variables. We show that landscapes with higher forest cover positively affected dung beetle body size. Interestingly, dung beetle responses to insularization were species‐dependent; larger islands tended to host larger individuals of *Deltochilum aspericole*, while in *Canthon triangularis*, smaller islands showed larger body sizes. However, individuals from the mainland were larger than those from the islands. Moreover, the proportion of closed‐canopy forest in the landscapes also impacted physiological attributes. It negatively affected the body size of *Deltochilum aspericole* and the lipid mass of *Dichotomius boreus*, but positively affected the lipid mass of *Canthon triangularis*. These findings contribute to a better understanding of how habitat fragmentation in aquatic matrices affects the size structure and physiology of insect assemblages. This is essential in formulating effective conservation strategies for preserving biodiversity loss in tropical forest regions and mitigating the consequences of hydropower infrastructure.

## Graphical abstract

Insular landscapes restrain dung beetles physiological condition. Dung beetles inhabiting riverine islands are smaller than their conspecifics from mainland. Insularization showed contrasting ecological responses, which differed depending on dung beetle species.

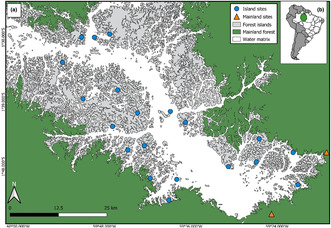

## 1 INTRODUCTION

The degradation and subdivision of previously intact landscapes into more isolated fragments have detrimental effects on the survival and persistence of species (Chase et al., 2020; Thomas, 2000). While most studies have focused on the ecological consequences of habitat fragmentation in remnants surrounded by croplands, cattle pastures, and urban matrices (e.g. Didham, 2010; Liu et al., 2016; Williams et al., 2006), few studies analyze such dynamics in landscapes dominated by aquatic matrix (e.g. Benchimol & Peres, 2015). Anthropogenic ‐caused insularization, characterized by the loss and fragmentation of otherwise continuous habitats, represents a pervasive threat to global biodiversity (Krauss et al., 2010). This phenomenon leads to increased habitat isolation, smaller habitat patches, reduced resource availability, and disruption of ecological processes due to the newly established fragmented landscapes (Didham, 2010). The presence of water as an impassable matrix act as an insurmountable barrier for most terrestrial species (Benchimol & Peres, 2015), exacerbating the challenges they face. Consequently, anthropogenic insularized sites (e.g. hydroelectric dams) comprise a unique landscape that can be used for assessing the effects of insularization on biological dynamics.

The construction of dams in the Amazon region has significant consequences for biodiversity, posing a challenge to its unique and complex ecological dynamics. Dams disrupt and alter otherwise continuous riparian ecosystems and adjacent habitats, leading to habitat isolation and the creation of fragmented ecosystems (Tullos et al., 2014). This disruption of natural landscape connectivity has far‐reaching impacts on species composition (Smith et al., 2017), population dynamics (Jellyman & Harding, 2012; Ngor et al., 2018), and ecological interactions (Zhu et al., 2021). Studying the physiological responses of species and whole assemblages in the context of shifts in landscape composition and structure is crucial for understanding the adaptive capacity and vulnerability of organisms to habitat fragmentation. Amazonian forest archipelagos created by large dams serve as an excellent, yet disquieting, model for studying the dynamics of insularization and its effects on the physiological condition of species and assemblages (Terborgh et al., 2001). By studying ecological communities that inhabit forest remnants within Amazonian hydropower reservoirs, it is possible to assess how changes in landscape structure and connectivity influence physiological processes, such as energy allocation and stress responses in aquatic matrices. Moreover, effective conservation strategies can be developed to mitigate any adverse consequences (Cooke et al., 2013), such as those imposed on Amazonian forest biodiversity due to the establishment of dams.

By the assessment of ecological processes under different biological scales, novel and wide insights can be drawn (Start & Gilbert, 2019). Individual‐species scale and the species assemblages scale has been used to present, respectively, finer and coarser understanding of ecological processes (Wellnitz et al., 2001). When analyzing species‐scale response, different approaches can be used, such as population structure (Laforge et al., 2016; Pecher et al., 2010), behavior (Leu et al., 2016; Pinaud & Weimerskirch, 2007) and physiological condition (Buckley et al., 2011; Cooke et al., 2013). Among these approaches, the physiological‐condition stands out as essential for comprehending the ecological dynamics associated with habitat changes. Through the study of specific physiological attributes such as lipid mass, muscle mass, and body dry mass, valuable insights can be obtained regarding how individuals, species, and assemblages adapt and respond to altered environmental conditions (França, Barlow, et al., 2016; França, Louzada, et al., 2016; Salomão et al., 2018, 2020). Moreover, physiological condition has consequences on species behavior and on the ecosystem services provided by them (Adolph, 1990; Amundrud & Srivastava, 2015; Salomão et al., 2021; Servín‐Pastor et al., 2021). This integrative approach, combining physiological and ecological perspectives at species and assemblage scale enhances our understanding of the complex relationships between organisms and their transforming habitats, thereby facilitating the development of effective conservation strategies.

Dung beetles (Coleoptera: Scarabaeinae) are excellent bioindicators of environmental disturbance (Correa et al., 2020; Halffter & Favila, 1993; Nichols et al., 2007; Noriega et al., 2021), responding to changes in biotic and abiotic attributes at both species and assemblage scales (França, Louzada, et al., 2016; Silva et al., 2019). Several features, such as their sensitivity to changes in environmental quality, along with simple and standardized sampling methods, make them excellent bioindicators (see Favila & Halffter, 1997). Studies have demonstrated that dung beetles undergo changes in assemblage attributes (Correa et al., 2020; Salomão, Alvarado, et al., 2019; Salomão, Favila, et al., 2019) and population structure (Barretto et al., 2023; Cultid‐Medina et al., 2015). More recently, individual physiological conditions (e.g. body dry mass, muscle mass, fat mass, see França, Barlow, et al., 2016; Salomão et al., 2018, 2020; Noriega et al., 2021) have gained relevance to understand the consequences of environmental changes on biodiversity. Regarding their physiological response towards habitat transformation, it has been observed that dung beetle physiological condition is negatively affected by forest disturbances such as selective logging (França, Barlow, et al., 2016), fragmentation (Salomão et al., 2018), and urbanization (Salomão et al., 2020). It is important to highlight that some ecological effects on biodiversity may be observed at physiological‐condition scale, but not when analyzing broader approaches, as population and assemblage structure (Cooke et al., 2013). Therefore, physical condition measurements can aid to predict how species respond to environmental changes (see Cooke et al., 2013; França, Barlow, et al., 2016; França, Louzada, et al., 2016; Salomão et al., 2020).

This study aimed to investigate the effects of insularization on the physiological condition and body size of dung beetles in a forest archipelago in the Central Amazon. Specifically, we assessed the effect of habitat type (island and continous forests), island size, shape, isolation, proximity, forest cover and the proportional area of closed‐canopy forest on body size, muscle mass, lipid mass, and body dry mass of individuals. To attain the goal of our study, we analyzed such dynamics under two ecological approaches: assemblage scale and species scale. The insularization processes generate changes in habitat availability, diversity, and quality in terms of resource availability and disturbance degree (Crouzeilles et al., 2014; Nyafwono et al., 2015; Welter‐Schultes & Williams, 1999).We therefore predict that body dry mass, muscle and lipid mass, and body size will be higher in larger, more connected islands with greater associated forest cover. For species‐scale approach, we used the most widely distributed dung beetle species in the region as model organisms: *Ateuchus murray* (Harold, 1868), *Canthon triangularis* (Drury, 1770), *Deltochilum aspericole* (Bates, 1870), *Dichotomius lucasi* (Harold, 1869), *and Dichotomius boreus* (Olivier, 1789). To our knowledge, this is the first assessment of the effects of insularization induced by a major hydroelectric dam on the physiological condition and body traits of any arthropod taxon.

## 2 MATERIALS AND METHODS

### 2.1 Study site

**FIGURE 1:**
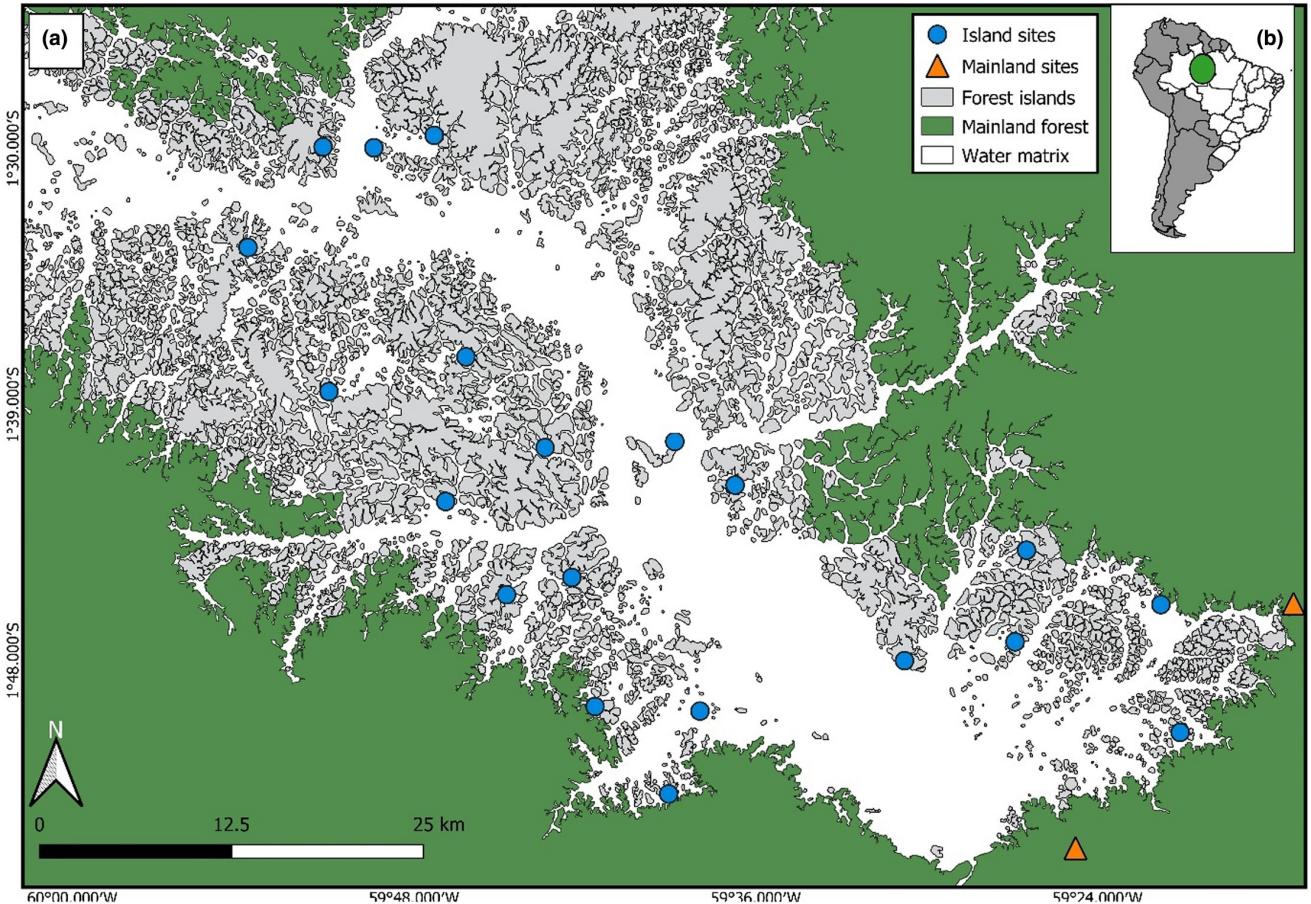
Map highlighting the region of the Amazonas state that encompasses the reservoir of the Balbina Hydroelectric Power Plant, with the studied islands (blue dots), and the mainland sites (oranged triangles) (a); South America, showing Brazil, (b).

The study was conducted in Balbina Hydroelectric Reservoir, located in the municipality of Presidente Figueiredo, Amazonas state, Brazil (1°52′S, 59°29′W) (See Figure 1). The reservoir was constructed in 1987, in the Uatumã River (a tributary of the Amazon River). With the closing of the Balbina dam, about 312 thousand hectares of tropical forest were flooded. Due to the topographically hilly characteristic of the area, the higher‐altitude regions turned into approximately 3500 islands that vary in size (0.2 ha to 4879 ha), shape, and distance from the nearest continuous forest (ranging from about 0.05 km to about 20 km of distance, Benchimol & Peres, 2015). The islands of this region are located from ca. 40 to 130 m a.s.l.

Currently, the group of islands forms a diverse landscape, with some islands retaining their original vegetation, while others have been affected by fires and wind (Aurélio‐Silva et al., 2016). The forests adjacent to the hydroelectric lake are classified as primary rainforests, ranging from ca. 90 to 180 m a.s.l. The climate of the region is defined as hot, humid, and rainy tropical (Am according to Köppen classification), presenting an average annual rainfall of 2376 mm, with a rainy season occurring from November to April (Peel et al., 2007; Walker et al., 1999). The average annual temperature varies around 28°C, and the average relative humidity remains around 97.2% throughout the year.

### 2.2 Sampling design

Sampling was carried out between April and May (end of the rainy season) in 2022. The study was conducted on 20 islands within the reservoir and two adjacent continuous forest areas, each study site spacing at least 1 km from each other, which guarantees spatial independence of each study site (Silva & Hernández, 2015). The islands were, on average, 4.72 km away from the mainland (See Figure 1 and Appendix S1). The islands were selected to form a gradient of size, degree of isolation from the nearest continuous forest, and forest coverage.

In each study site, pitfall traps were used to collect dung beetles. Pitfall traps consisted of a cylindrical plastic container of 500 mL with a smaller bait‐container plastic cup (50 mL) installed ca. 3 cm above the larger plastic container. A suspended plastic lid was installed over the trap to prevent the entry of leaves, branches, and rainwater. To preserve the collected material for physiological measurements, 70% ethanol was placed inside the container. Ten traps were installed in each study site (i.e., in each island and in each continuous forest area), baited with approximately 25 g of fresh human feces. To maximize and standardize the sampling effort, each trap was placed at least 20 m apart from each other (adapted from Salomão et al., 2021). All traps were active in field for 48 h. The collected individuals were taken to National Institute of Amazonian Research (INPA) laboratories, where body size and physiological condition were obtained.

### 2.3 Landscape variables

The landscape variables were obtained from Storck‐Tonon et al. (2020). Therefore, using a seamless RapidEye mosaic (5 m pixel resolution) of georeferenced satellite imagery we obtained the island area (ha); island forest cover (%); the proportional area (%) of closed‐canopy forest, and island shape. Island shape was calculated using an index that comprises the ratio of the perimeter to the area of each. In addition, we also obtained a proximity index (proximity among islands within the 250, 500, and 1000 m) buffer and isolation index (isolation distance (m) from the nearest mainland site). The landscape variables were calculated using circular landscapes (buffers) of 250, 500, and 1000 m radius. These different scales will be used since the responses of tropical dung beetles vary according to the analyzed scale (e.g., Salomão et al., 2020; Salomão, Alvarado, et al., 2019; Salomão, Favila, et al., 2019). For more information on our landscape variables, see (Storck‐Tonon et al., 2020).

### 2.4 Physiological condition and body size

Three indicators of physiological condition were used: body dry mass, lipid mass, and muscle mass. Body dry mass directly reflects individuals' fitness (Briffa & Sneddon, 2007; Córdoba‐Aguilar et al., 2016); lipid mass represents the amount of energy reserves of individuals (Schulte‐Hostedde et al., 2005); muscle mass is directly related to reproduction (Marden & Cobb, 2004) since it approximates courtship vigor and testicular mass (Droney & Hock, 1998). Following the procedures of Lee et al. (2004), to estimate body dry mass (i.e., dry weight), beetles were dried in a 50°C oven for 48 h. Then, each individual was weighed using a Mettler Toledo AB265‐S precision balance with a resolution of 0.0001 g. Next, lipids were extracted by placing the individual dried beetles in containers containing 2 mL of chloroform for 24 h, twice in a row. After this period, the beetles were dried (at the same time and temperature used previously) and weighed again. The difference between dry weight and the weight of the beetle after lipid extraction was considered as lipid mass. For muscle mass measurements, the procedures of Baines et al. (2015) and Marden (1989) were adjusted. After lipid mass was determined, beetles were placed in 2 mL of 0.8 M KOH for 48 h, rinsed, dried, and weighed again. The difference between the weight without lipid mass and this new weight was considered muscle mass. Body size was estimated from the widest linear distance of the pronotum margins (horizontal line in relation to the longitudinal axis of the individual) (Salomão et al., 2018). Body size was measured using digital images taken through an AxioCam ICc 3 camera attached to a ZEISS SteREO Discovery.V12 stereomicroscope.

### 2.5 Data analyses

To identify the spatial scale (250, 500, and 1000 m of radius) at which each landscape variable best explains our response variables, we used the area‐landscape approach proposed by Fahrig (2013). For our analyses, we only used the scale of landscapes with highest *R* ^2^ values (Fahrig, 2013). This approach has been previously used in ecological studies with tropical dung beetle assemblages (see Salomão et al., 2020; Salomão, Alvarado, et al., 2019; Salomão, Favila, et al., 2019).

We used linear mixed models (LMM) and generalized linear models (GLM) to analyze the effects related to insularization on the physiological condition and body size of dung beetles. As predictor variables, we used the landscape variables and the type of habitat (island or mainland). As response variable, we used individuals' body dry mass, lipid mass, muscle mass, and body size. For the variables body dry mass, lipid mass, and muscle mass, the values were relativized by the individuals' body size (i.e., individual body masses were divided by body size). To observe the magnitude of the effects of insularization on the physiological condition and body size of the beetles, we analyzed all species simultaneously (assemblage scale) and for each one separately (species scale). At the species scale, GLMs were performed with Gaussian distribution (which is equivalente to linear models) and at assemblage scale, LMMs were performed with the species identity being considered as a random variable. At species scale, only the species that we collected more than 10 individuals and in more than 7 sites were selected. Residual normality, homoscedasticity, and presence of outliers were checked using the DHARMa package (Hartig, 2022). When variances were heterogeneous, different variance structures were tested. In order to use the predictor variables that best explained the distribution of dung beetles' physiological condition and body size, we performed model selection (Johnson & Omland, 2004). The best‐supported model was selected based on the Akaike Information Criterion (AIC) value (Zuur et al., 2009), by the stepAIC function of the MASS package (Venables & Ripley, 2002). We used conditional graphs, via the visreg package (Breheny & Burchett, 2017), to visualize the fit of the regression model, which show the variation in the response variable (partial residual) in relation to the predictor variables alone (Breheny & Burchett, 2017). All analyses were performed using R software version 4.2.1 (R Core Development Team, 2020).

## 3 RESULTS

A total of 321 individuals belonging to 20 species were collected (see Appendix S1). The island with the highest abundance (Jabuti) comprised 37 individuals from six species. On the other hand, the three least abundant islands (Bacaba, Fuzarca, and Pé Torto) had only one individual each, belonging to *D. subaenaeus*, *D. lucasi*, and *C. triangularis*, respectively (Appendix S1). The most abundant species in island habitats were also the most widely distributed species (*C. triangularis – n* = 66, being recorded in six islands; *A. murrayi – n* = 51, recorded in seven islands). Interestingly, all 71 individuals of *Ateuchus simplex* (Le Peletier & Serville, 1828) were collected only from the mainland. Since species‐level analyses of insularization effects on individuals' physiological condition and body size needed species recorded in at least 7 sites for a miminum statistical trustworthiness, *A. simplex* was not included in these analyses. The rarest species, *Uroxys* (Westwood, 1842) sp. and *Deltochilum submetallicum* (Castelnau, 1840), were each represented by only one individual.

The largest and heaviest (i.e. highest body dry mass) species were *D. boreus* and *D. subaenaeus* (Table 1) and the smallest and lightest specie were *Uroxys* sp. (Table 1). Among the species used to analyze insularization effects at the species‐scale approach, *D. boreus* was the largest and heaviest and *A. murrayi* was the smallest and lightest (Table 1). Also, according to the species used in the species‐scale approach, *D. boreus* was the one with highest relative fat mass, while *Deltochilum aspericole* was the one with lowest fat mass (Table 1). *Deltochilum submetallicum* was the one with highest relative muscle mass, and *D. boreus* was the one with lowest relative muscle mass (Table 1).

**TABLE 1:** Abundance, body size, body dry mass, relative lipid mass, and relative muscle mass of the species collected.

Species	Body size (mean ± SD mm)	Body dry mass (mean ± SD g*10)	Relative lipid mass (mean ± SD g/mm*10^3^)	Relative muscle mass (mean ± SD g/mm*10^3^)
*Ateuchus cereus* (*n* = 11)	2.01 ± 0.17	0.03 ± 0.01	NA	NA
*Ateuchus globulus* (*n* = 4)	2.27 ± 0.07	0.04 ± 0.01	NA	NA
*Ateuchus murrayi* (*n* = 51)	2.69 ± 0.19	0.01 ± 0.02	NA	NA
*Ateuchus simplex* (*n* = 71)	3.88 ± 0.16	0.20 ± 0.04	NA	NA
*Ateuchus* sp. (*n* = 1)	NA	NA	NA	NA
*Canthidium deyrollei* (*n* = 1)	NA	NA	NA	NA
*Canthon sordidus* (*n* = 5)	4.24 ± 0.80	0.20 ± 0.04	NA	NA
*Canthon triangularis* (*n* = 66)	5.57 ± 0.20	0.40 ± 0.10	0.65 ± 0.53	3.57 ± 1.31
*Coprophanaeus jasius* (*n* = 3)	NA	NA	NA	NA
*Coprophanaeus lancifer* (*n* = 1)	NA	NA	NA	NA
*Deltochilum aspericole* (*n* = 11)	5.42 ± 0.17	0.40 ± 0.10	0.45 ± 0.38	2.30 ± 1.49
*Deltochilum icarus* (*n* = 1)	NA	NA	NA	NA
*Deltochilum submetallicum* (*n* = 1)	7.19 ± 0.00	1.70 ± 0.00	2.77 ± 0.00	9.09 ± 0.00
*Dichotomius boreus* (*n* = 15)	15.16 ± 1.55	6.70 ± 2.20	6.04 ± 3.82	1.53 ± 2.80
*Dichotomius lucasi* (*n* = 48)	7.56 ± 0.37	1.00 ± 0.40	2.10 ± 2.07	2.53 ± 1.64
*Dichotomius subaenaeus* (*n* = 20)	9.64 ± 1.12	2.20 ± 0.80	3.94 ± 3.76	4.24 ± 2.88
*Eurysternus atrosericus* (*n* = 5)	NA	NA	NA	NA
*Eurysternus caribaeus* (*n* = 4)	NA	NA	NA	NA
*Ontherus* sp. (*n* = 1)	NA	NA	NA	NA
*Uroxys* sp. (*n* = 1)	1.59 ± 0.00	0.01 ± 0.00	NA	NA

### 3.1 Assemblage‐scale effects of insularization

**FIGURE 2:**
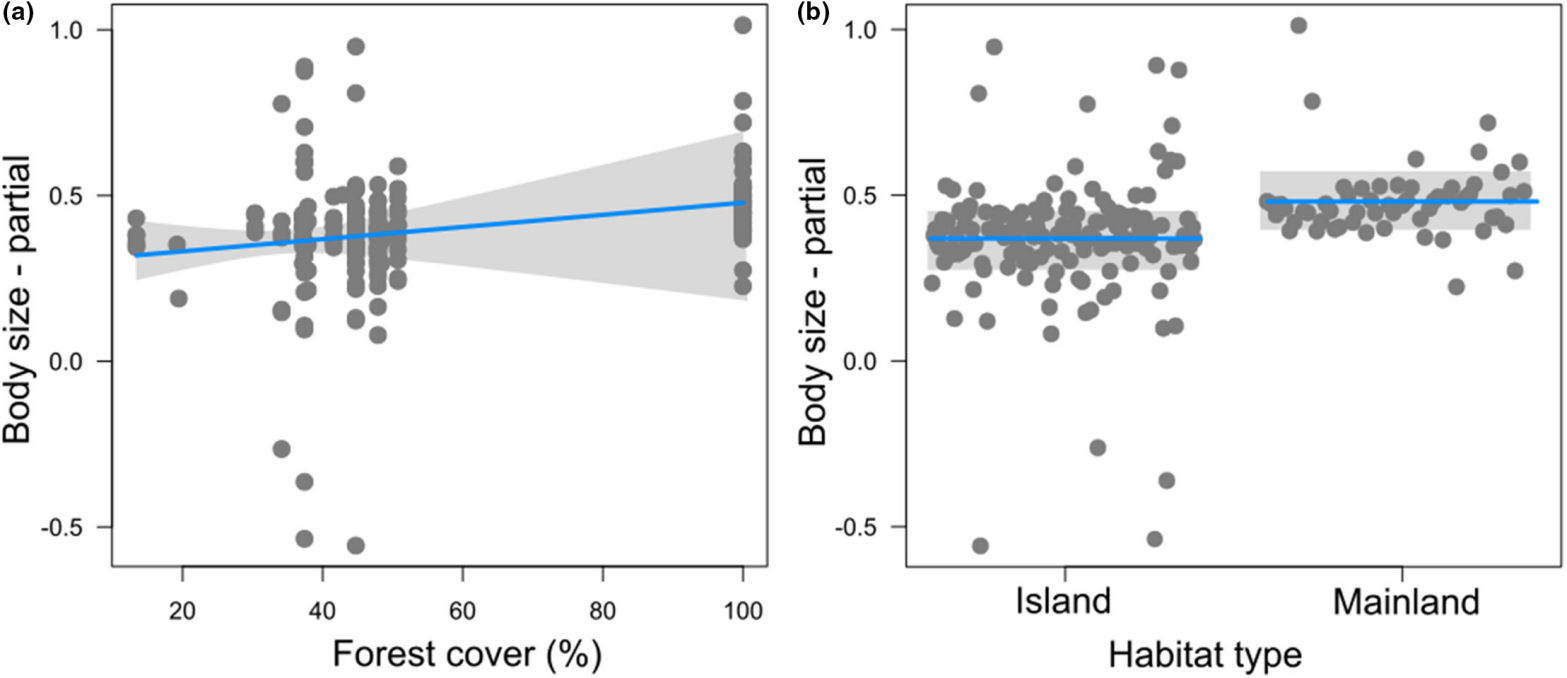
Partial regression plots showing the assemblage‐scale effects of forest cover (buffer 500 m) (a) and habitat type (b) on body size. Lines represent model predictions of statistically supported effects (a) and mean (b); gray bands indicate 95% confidence intervals, and partial residuals are given by dots.

The forest cover affected body size of dung beetle assemblages. Landscapes with higher forest cover encompassed dung beetles with larger body sizes (Figure 2a). Regarding habitat type (island and mainland), individuals from the mainland have larger body size than those from the islands (Figure 2b). However, habitat type did not affect dung beetles' body masses. Similarly, island shape, proximity, the proportion of closed canopy forest, island area and isolation did not affect individuals' body size and body masses.

### 3.2 Species‐scale effects of insularization

**FIGURE 3:**
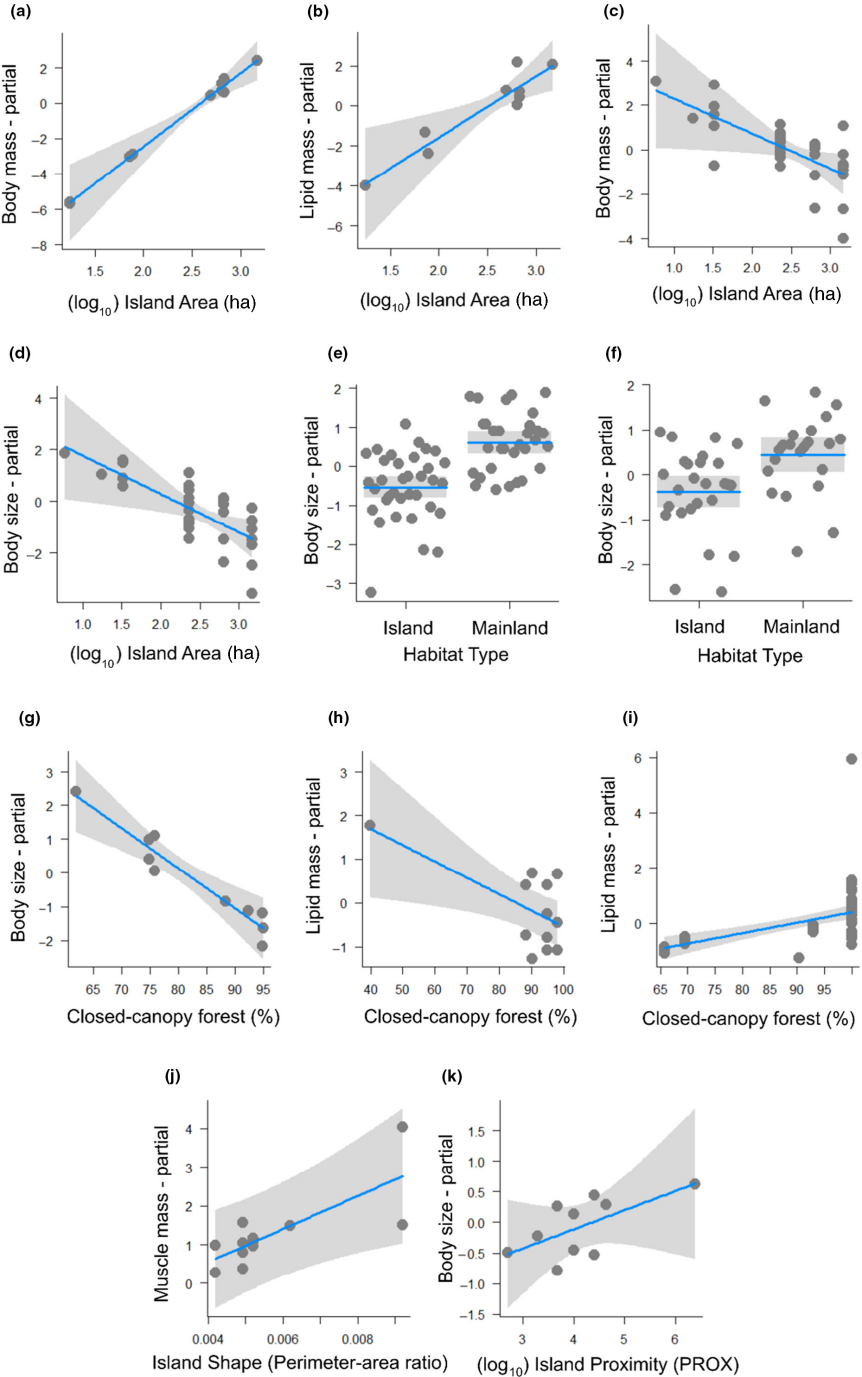
Partial regression plots of the effects of island area on standardized body dry mass of *Deltochilum aspericolle* (a); island area on standardized lipid mass (b) of *Deltochilum aspericolle*; island area on standardized body dry mass (c) of *Canthon triangularis*; island area on body size (d) of *Canthon triangularis*; habitat type on body size (e) of *Canthon triangularis*; and habitat type on body size (f) of *Dichotomius lucasi*; closed‐canopy forest (buffer 250 m) (g) on body size of *Deltochilum aspericolle*; closed‐canopy forest (buffer 250 m) on standardized lipid mass (h) of *Dichotomius boreus*; closed‐canopy forest on standardized lipid mass (i) of *Canthon triangularis*; island shape on standardized muscle mass (j) of *Dichotomius boreus*; island proximity (k) on body size of *Deltochilum aspericolle*. Lines represent model predictions of statistically supported effects and mean (e, f); gray bands indicate 95% confidence intervals, and partial residuals are given by dots. Standardized masses means that the values were adjusted for body size (for a more detailed information regarding the estimation of body masses, see materials and methods).

Insularization effects on the studied dung beetle species are summarized in Table 2. Larger islands encompasses individuals with higher body dry mass (Figure 3a) and lipid mass (Figure 3b) in *D. aspericolle*, but also encompassed individuals with lower body dry mass (Figure 3c) and body size in *C. triangularis* (Figure 3d). When comparing habitat types, mainland individuals of *C. triangularis* and *D. lucasi*, were statistically larger than those conspecifics recorded in islands (Figures 3e,f, respectively). Landscapes with higher percentage of closed‐canopy forest dwelled smaller‐bodied populations of *D. aspericolle* (Figure 3g), and lower lipid mass in the largest species, *D. boreus* (Figure 3h). Nonetheless, the percentage of closed‐canopy forests had a positive relationship with lipid mass in *C. triangularis* individuals (Figure 3i). Islands with higher perimeter‐to‐area ratio present individuals with higher muscle mass in *D. boreus* individuals (Figure 3j). Landscapes with higher island proximity dwelled larger individuals of *D. aspericolle* (Figure 3k).

**TABLE 2:** Results of the statistical models that analyzed the influence of predictive variables on the physiological condition and body size of dung beetles at both the species and assemblage levels.

	Habitat type		Forest cover		Closed‐canopy Forest		Shape index (per/area)		Proximity		Island area		Isolation	
**Species‐scale effects**														
*Deltochilum aspericolle*														
Body size	*F*2 = 0.38	*p* = .55	NS		***F*1 = 15.77**	***p* = .01**	*F*1 = 4.85	*p* = .08	***F*1 = 10.54**	***p* = .02**	NS		*F*1 = 1.44	*p* = .28
Body dry mass	*F*2 = 1.74	*p* = .22	*F*1 = 7.09	*p* = .80	*F*1 = 8.85	*p* = .06	*F*1 = 2.08	*p* = .25	*F*1 = 2.85	*p* = .19	***F*1 = 43.53**	***p* = .01**	*F*1 = 0.80	*p* = .44
Lipid mass	*F*2 = 0.87	*p* = .38	NS		NS		*F*1 = 2.19	*p* = .18	NS		***F*1 = 8.78**	***p* = .02**	NS	
Muscle mass	*F*2 = 1.81	*p* = .21	*F*1 = 1.44	*p* = .30	NS		*F*1 = 0.01	*p* = .92	*F*1 = 5.40	*p* = .08	*F*1 = 6.00	*p* = .07	*F*1 = 2.36	*p* = .20
*Dichotomius boreus*														
Body Size	*F*2 = 3.67	*p* = .08	NS		NS		*F*1 = 3.73	*p* = .08	NS		NS		NS	
Body dry mass	*F*2 = 0.85	*p* = .37	NS		*F*1 = 0.33	*p* = .58	NS		NS		NS		NS	
Lipid mass	*F*2 = 0.02	*p* = .91	NS		***F*1 = 6.08**	***p* = .04**	*F*1 = 4.08	*p* = .07	NS		NS		NS	
Muscle mass	*F*2 = 0.37	*p* = .56	*F*1 = 0.20	*p* = .67	*F*1 = 3.29	*p* = .11	***F*1 = 8.77**	***p* = .02**	*F*1 = 0.59	*p* = .47	NS		NS	
*Dichotomius lucasi*														
Body size	***F*2 = 9.37**	***p* < .01**	NA		NA		NA		NA		NA		NA	
Body dry mass	*F*2 = 0.37	*p* = .55	NA		NA		NA		NA		NA		NA	
Lipid mass	*F*2 = 1.08	*p* = .30	NA		NA		NA		NA		NA		NA	
Muscle mass	*F*2 = 0.88	*p* = .35	NA		NA		NA		NA		NA		NA	
*Canthon triangularis*														
Body size	***F*2 = 31.17**	***p* < .01**	NS		NS		*F*1 = 0.96	*p* = .33	NS		***F*1 = 6.78**	***p* = .01**	NS	
Body dry mass	*F*2 = 2.55	*p* = .12	NS		NS		*F*1 < 0.01	*p* = .95	NS		***F*1 = 4.72**	***p* = .04**	NS	
Lipid mass	*F*2 = 0.77	*p* = .39	NS		***F*1 = 25.5**	***p* < .01**	NS		NS		NS		NS	
Muscle mass	*F*2 = 0.16	*p* = .69	NS		NS		NS		NS		NS		NS	
*Ateuchus murrayi*														
Body size	NA		NS		*F*1 = 3.14	*p* = .08	NS		NS		NS		NS	
Body dry mass	NA		NS		*F*1 = 3.19	*p* = .08	NS		NS		NS		NS	
**Assemblage‐scale effects**														
Body size	***t* = 3.68**	***p* < .01**	***t* = 3.63**	***p* < .01**	NS		NS		NS		NS		NS	
Body dry mass	*t* = 0.78	*p* = .44	NS		*t* = 0.35	*p* = .73	NS		*t* = −0.19	*p* = .85	NS		NS	
Lipid Mass	*t* = − 0.68	*p* = .50	NS		NS		NS		*t* = −0.58	*p* = .56	NS		NS	
Muscle mass	*t* = 0.46	P = .65	NS		NS		*t* = 0.81	*p* = .42	*t* = −1.5	*p* = .14	*t* = 0.89	*p* = .38	NS	

*Note*: Variables that were statistically significant are shown in bold. NA, Variables not applied in the model; NS, variables not selected by the best‐supported model. *F* values refers to Fishers statistics. Physiological condition was estimated by standardized masses, which were obtained by dividing body masses by individual body sizes (for a more detailed information regarding the estimation of body masses, see materials and methods).

The landscape scale that best explained the distribution of response variables in the following result sections are included in the Appendix S1. Statistical effects of insularization, both at assemblage scale and species scale, are shown in Table 2. The variables not presented in the following statistical results were not included in the best model following the AIC criteria (see ‘variables not selected by the best‐supported model’ in Table 2).

## 4 DISCUSSION

In this study, we explored how insularization in the Amazonian forest has affected the body size and physiological condition of dung beetles 36 years after the creation of this human‐made insular scenario caused by flooding in the Amazonian region. We analyzed these fitness‐related traits of dung beetles under a landscape and habitat type perspective. At the species scale, island area and the proportional amount of closed‐canopy forests emerged as the most determinant variables for body size and physiological condition of dung beetles. At the assemblage scale, habitat type, and forest cover featured the most influential variables. Species of dung beetles responded differently to landscape metrics, emphasizing the importance of considering species‐ specific responses when assessing ecological dynamics. By focusing on the physiological condition and body traits of dung beetles, our study offers a reliable proxy of how isolation and landscapes within islands impact the health and fitness of individual organisms in one of the most diverse regions in the world. Our results indicated that body size and physiological condition of the dung beetles vary between the islands and mainland. Such findings provided novel insights into landscape ecology, demonstrating the complex interplay between landscape metrics and assemblage‐species‐scale traits in shaping the body size and physiological condition of dung beetles.

### 4.1 Island area as an important landscape‐scale driver of dung beetle physiology and body size

The amount of habitat can affect the availability of food resources, microclimatic conditions, and habitat structure in tropical rainforests (Karanth & Sunquist, 2000; Kessler et al., 2005; Nyafwono et al., 2015), which are known to influence dung beetle metabolism, energy balance, and body traits (Batilani‐Filho & Hernández, 2017; Feer, 2013; Kerley et al., 2018; Salomão et al., 2018). Our study found that island area had a positive relationship with the body dry mass and lipid mass of *D. aspericolle*, but had a negative relationship with the body dry mass and body size of *C. triangularis*. Studies have shown that larger islands dwell higher mammal species richness and abundance (Neto et al., 2022; Palmeirim et al., 2018), which are the main providers of food resources for dung beetles (Nichols et al., 2008). Our results partially support the idea that larger islands offer more resources and more stable habitats, since *D. aspericolle* (but not *C. triangularis*) presented proxies of such trend. Under conditions of low food availability, such as on small islands, dung beetles may face trade‐offs between allocating energy to processes such as reproduction and growth or investing energy on vital physiological functions such as maintenance and repair (Kooijman, 1986; Stearns, 1989). Such trade‐offs might differ between males and females, which have different strategies of resource acquisition and use: whereas females use their energetic budget for egg production and self‐maintenance, males invest energy in finding and monopolizing mates, even sacrificing self‐care (Boggs, 2009; Fanson et al., 2013; Lease & Wolf, 2011). Further analyses of male and female strategies of resource intake and use could sheld light on how both sexes respond to insularization. Moreover, analyses of sex ratios in different islands could indicate to what extent mate and food availability can define migration between islands and cause differences in the physiological condition of both sexes (Carmona‐Isunza et al., 2017).

Competition for resources is another factor that can significantly impact animal fitness, particularly in habitats where resources are scarce or where species are abundant (Hanski, 1991; Rodenhouse et al., 2003; Sillett et al., 2004). Competition among individuals for access to those resources can lead to changes in the physiological and morphological characteristics of animals over time (Svanbäck & Bolnick, 2005; Svanbäck & Bolnick, 2007; Yund, 1991). Although competition and nutrient availability are key factors in determining individual physiological condition, it is still uncertain what mechanisms could have driven the opposite patterns observed in *C. triangularis* and *D. aspericolle* beetles. Natural history and species traits (e.g., dial activity flight, perching, and nesting behavior) may give us future cues of how each species uses and is affected by landscape parameters in disturbed forests.

### 4.2 Habitat type effects

We found that individuals from the mainland were larger than those from islands, but physiological condition was not affected by habitat type. Whereas body size is determined during larval development, physiological condition is sensitive to current conditions (Baines et al., 2015; Karino et al., 2004; Moczek, 1998). Thus, our results indicate that insularization effects are not similar during larval development and adult stage. Indeed, insect larvae feeding on high amount of good‐quality resources emerge as large adults (Karino et al., 2004; Moczek, 1998), which may indicate, in a first moment, that resource availability/quality for larval development was different between mainland and islands. Body size is determinant for animal fitness, with large‐bodied individuals tending to present higher fecundity (in females) and mating success (in males) than smaller ones (Arnott & Elwood, 2009; Chamorro‐Florescano et al., 2011; Nosil, 2002). In addition, larger beetles could have advantages in food competition, obtaining food more successfully, and therefore could choose the most valuable resource (i.e. the most nutritious one or the best resource for breeding) (Solomon et al., 2019). Thus, we may expect that intra‐ and interspecific competition dynamics within dung beetle assemblages could differ between mainland and the islands of the current ecosystem. Curiously, physiological condition trends were scale‐dependent, with clear relationships between landscape metrics and physiological condition, but the absence of the effect of habitat type. The absence of habitat effect on physiological condition may indicate that the different island properties (e.g. landscape metrics, plant and animal diversity, vegetation structure) could blurry the potential consequences of insularization. Such trend is observed in ecological studies in tropical ecosystems (Douda, 2010; Hernández‐Stefanoni et al., 2011; Lomba et al., 2011), thus highlighting the importance to locate the best scale to explain ecological processes. An alternative hypothesis is that the smaller‐bodied populations that dwells in islands could present lower energetical needs, therefore buffering physiological consequences of insularized landscapes on individual physiological condition.

It is important to consider that we are unaware of the dispersion patterns of dung beetles throughout islands. Although we do not have clear cues regarding this spatial dynamics, there was reduced abundance and diversity of dung beetles in island compared to mainland (which was observed herein and in Salomão et al., 2020). The low abundance in islands compared to mainland could suggest that dung beetles are not moving among islands, or that only a reduced number of individuals could be moving among them. Regarding this second hypothesis, this could be specially true in the islands which had only one individual collected. These records could be species passing through, but only some of these islands could be permanent habitat. As observed in previous studies, forest‐dweller dung beetles have highly restrained distributions in terrestrial open matrices, as sugarcane plantations and pasturelands (Alonso et al., 2020; Filgueiras et al., 2015). However, it is unclear how dung beetles disperse across the water matrix. To attain a deeper comprehension of the findings of our research, future mark‐recapture studies (Barretto et al., 2023; Cultid‐Medina et al., 2015) should assess how water limits dung beetle dispersal and survival rates in islands. By shedding light on these issues, a more complex source‐sink population dynamics can be drawn in insular tropical landscapes.

### 4.3 Forest cover did not affect dung beetles' physiological condition

While the crucial role of forest cover in determining biodiversity in tropical ecosystems has been widely recognized (Alvarado et al., 2018; Arroyo‐Rodríguez et al., 2016; Galán‐Acedo et al., 2019; Noriega et al., 2021; Watling et al., 2020), our study sheds light on the context‐dependent nature of its influence. Specifically, our investigation of the Amazonian archipelago reveals that the impact of forest cover on biodiversity may differ in this unique ecological setting compared to others landscapes. When compared to other fragmented rainforest landscapes (e.g. Atlantic Forest), the Amazonian archipelago have different characteristics and ecological dynamics that set it apart. Possibly, one of the main differences is the water matrix, comprising a physical barrier for most animals and plants. The presence of islands, as well as factors like island area and proportion of closed‐canopy forest, introduces additional complexities to the relationship between forest cover and biodiversity. Considering that matrix type is a determinant factor for forest fragmentation dynamics (Jules & Shahani, 2003; Prevedello & Vieira, 2010), we believe that the water‐matrix fragmented landscape that we studied herein may play a key role for ecological dynamics. This hypothesis is reinforced by the previous study with dung beetle assemblages in the same Amazonian archipelago (Storck‐Tonon et al., 2020). Compared to rainforest patches in plantation or pastureland matrices (e.g. Filgueiras et al., 2015; Quintero & Roslin, 2005; Rodríguez‐García et al., 2021), the forest fragments in a water matrix maintain astonishingly poor species richness and abundance of dung beetles (Storck‐Tonon et al., 2020). Interestingly, our findings suggest that landscape metrics other than forest cover exert a stronger influence on the physiology and body size of dung beetles. Consequently, in the Amazonian archipelago, the relative importance of forest cover in determining dung beetles' physiology may be overshadowed by the effects of the water‐matrix scenario. These factors likely play a more dominant role in shaping the ecological dynamics and characteristics of dung beetle assemblages in this specific context.

It is important to recognize that our findings do not diminish the overall importance of forest cover for biodiversity conservation in tropical ecosystems. Forest cover remains as a fundamental factor for the maintenance of ecosystem stability, as it provides habitat, resources, and ecosystem services to a wide range of species (Lee et al., 2007; López‐Bedoya et al., 2022; Solomon et al., 2019; Zellweger et al., 2020). However, in the case of the Amazonian archipelago, other environmental factors related to island dynamics and microclimatic conditions, such as island area and the proportion of closed‐canopy forest, may exert a stronger influence on dung beetles' physiology. To gain a comprehensive understanding of the complex relationships between forest cover, environmental factors, and biodiversity in the Amazonian archipelago, further research is needed. Future studies could explore the specific mechanisms through which island characteristics and microclimatic conditions interact with forest cover to shape dung beetle health and fitness.

### 4.4 Species‐specific contrasting physiological response to insularization

One of the key results of this study comes from the specific and contrasting responses presented by each dung beetle species. Species‐specific physiological responses may be related to ecological requirements (e.g. temperature conditions and landscape configuration) and life history strategies of each species (Salomão et al., 2018; Williamson et al., 2022). Our study reveals that the impact of the proportion of closed‐canopy forests on dung beetle species is complex, with contrasting effects on the studied species. Specifically, we found a negative relationship between the proportion of closed canopy and the lipid mass of *D. boreus* individuals, but a positive relationship with the lipid mass of *C. triangularis* individuals. It is possible that each species has developed different ecological strategies in response to light, temperature, and humidity, which are influenced by closed canopy coverage. For example, *D. boreus* may gain a competitive advantage in open environments, benefiting from its adaptability as a nocturnal species. Interestingly, a study by Barretto et al. (2021) showed that a species of the genus *Dichotomius* exhibited reduced activity within forested areas and displayed higher mobility in non‐forest areas. Conversely, *C. triangularis* may thrive in areas with closed canopy coverage, utilizing the canopy as a resource due to its diurnal behavior. Canopy coverage can have several potential effects on shelter, temperature regulation, and resource utilization by different species (Chen et al., 1999; Natsukawa et al., 2019;). Canopy coverage provides protection against direct sunlight, which is crucial for diurnal species sensitive to dehydration and excessive heat (Gotcha et al., 2020). Moreover, it can reduce visibility to potential predators, such as birds (Young, 2015), increasing the safety of the dung beetles inhabiting the understory. Species that perch in the canopy can access resources, such as feces found on leaves, that would otherwise be out of reach for non‐perching species (Rahman et al., 2021). Therefore, if *C. triangularis* happens to be one of the few species that perch or fly high in the canopy, it can potentially exploit resources that others cannot access. Our findings underscore the importance of understanding species‐specific responses when assessing the impact of environmental changes on biodiversity and emphasize the need for further research on the physiological implications of habitat transformation.

### 4.5 Conclusion

Insularization effects on animal physiology remain largely untested and deserve further attention in organisms that play key roles in maintaining ecosystem functioning, such as dung beetles. Our results indicate that islands are more restrictive habitats for dung beetles than continuous forests. This study highlights the importance of categorizing systems to obtain a more comprehensive understanding of how environmental transformations affect species and community responses. It is worth noting that forest cover, isolation, island area, and the amount of closed canopy forests plays a crucial role in determining biodiversity in tropical insular ecosystems. Nonetheless, our results were contrasting and species‐dependent, providing a complex set of cause and consequences between landscape variables and assemblage and species physiological condition. Species‐dependent results are an irrefutable indicator that each species has specific requirements. This type of information is of utmost importance for understanding the processes that occur at the individual and population scales, as well as when establishing a conservation strategy.

## AUTHOR CONTRIBUTIONS

**Leonardo Vilas‐Bôas M. P. de Cerqueira:** Conceptualization (equal); data curation (equal); formal analysis (lead); investigation (equal); methodology (equal); writing – original draft (lead); writing – review and editing (equal). **Daniel González Tokman:** Methodology (equal); supervision (equal); writing – review and editing (equal). **César M. A. Correa:** Writing – review and editing (equal). **Danielle Storck‐Tonon:** Investigation (equal); resources (equal). **Mario Cupello:** Data curation (equal). **Carlos A. Peres:** Conceptualization (equal); resources (equal); writing – review and editing (equal). **Renato Portela Salomão:** Conceptualization (equal); data curation (equal); funding acquisition (lead); investigation (equal); methodology (equal); project administration (lead); writing – original draft (equal); writing – review and editing (lead).

## CONFLICT OF INTEREST STATEMENT

The authors declare that they have no known competing financial interests or personal relationships that could have appeared to influence the work reported in this paper.

## Supporting information


Appendix S1.

## Data Availability

The data that supports the findings of this study are available in the Appendix S1 of this article.
